# The synthetic triterpenoid CDDO-methyl ester modulates microglial activities, inhibits TNF production, and provides dopaminergic neuroprotection

**DOI:** 10.1186/1742-2094-5-14

**Published:** 2008-05-12

**Authors:** Thi A Tran, Melissa K McCoy, Michael B Sporn, Malú G Tansey

**Affiliations:** 1 Department of Physiology, The University of Texas Southwestern Medical Center, Dallas, Texas, USA; 2 Department of Pharmacology, Dartmouth Medical School, Hanover, New Hampshire, USA

## Abstract

**Background:**

Recent animal and human studies implicate chronic activation of microglia in the progressive loss of CNS neurons. The inflammatory mechanisms that have neurotoxic effects and contribute to neurodegeneration need to be elucidated and specifically targeted without interfering with the neuroprotective effects of glial activities. Synthetic triterpenoid analogs of oleanolic acid, such as methyl-2-cyano-3,12-dioxooleana-1,9-dien-28-oate (CDDO-Me, RTA 402) have potent anti-proliferative and differentiating effects on tumor cells, and anti-inflammatory activities on activated macrophages. We hypothesized that CDDO-Me may be able to suppress neurotoxic microglial activities while enhancing those that promote neuronal survival. Therefore, the aims of our study were to identify specific microglial activities modulated by CDDO-Me *in vitro*, and to determine the extent to which this modulation affords neuroprotection against inflammatory stimuli.

**Methods:**

We tested the synthetic triterpenoid methyl-2-cyano-3,12-dioxooleana-1,9-dien-28-oate (CDDO-Me, RTA 402) in various *in vitro *assays using the murine BV2 microglia cell line, mouse primary microglia, or mouse primary peritoneal macrophages to investigate its effects on proliferation, inflammatory gene expression, cytokine secretion, and phagocytosis. The antioxidant and neuroprotective effects of CDDO-Me were also investigated in primary neuron/glia cultures from rat basal forebrain or ventral midbrain.

**Results:**

We found that at low nanomolar concentrations, treatment of rat primary mesencephalon neuron/glia cultures with CDDO-Me resulted in attenuated LPS-, TNF- or fibrillar amyloid beta 1–42 (Aβ1–42) peptide-induced increases in reactive microglia and inflammatory gene expression without an overall effect on cell viability. In functional assays CDDO-Me blocked death in the dopaminergic neuron-like cell line MN9D induced by conditioned media (CM) of LPS-stimulated BV2 microglia, but did not block cell death induced by addition of TNF to MN9D cells, suggesting that dopaminergic neuroprotection by CDDO-Me involved inhibition of microglial-derived cytokine production and not direct inhibition of TNF-dependent pro-apoptotic pathways. Multiplexed immunoassays of CM from LPS-stimulated microglia confirmed that CDDO-Me-treated BV2 cells produced decreased levels of specific subsets of cytokines, in particular TNF. Lastly, CDDO-Me enhanced phagocytic activity of BV2 cells in a stimulus-specific manner but inhibited generation of reactive oxygen species (ROS) in mixed neuron/glia basal forebrain cultures and dopaminergic cells.

**Conclusion:**

The neuroimmune modulatory properties of CDDO-Me indicate that this potent antioxidant and anti-inflammatory compound may have therapeutic potential to modify the course of neurodegenerative diseases characterized by chronic neuroinflammation and amyloid deposition. The extent to which synthetic triterpenoids afford therapeutic benefit in animal models of Parkinson's and Alzheimer's disease deserves further investigation.

## Background

Plant-derived triterpenoids, including oleanolic acid and ursolic acid, have been used extensively in Asian countries for their anti-inflammatory and anti-tumor properties [1]. In an attempt to increase the potency of these natural products, over 300 synthetic derivatives of oleanolic acid were generated and tested for their ability to inhibit NO production in activated macrophages [2-4]. Some of the most potent of these, including 2-cyano-3,12-dioxooleana-1,9-dien-28-oic acid (CDDO; RTA 401) and its methyl ester (CDDO-Me; RTA 402), exhibit greater than 2 × 10^5^-fold increased potency compared to the parental compound. CDDO-Me is presently in Phase I/II clinical trials for the treatment of solid tumors. In light of their potent bioactivity, this new class of compounds has therapeutic potential in the treatment and prevention of acute and chronic inflammatory syndromes.

Although identification of the molecular targets of triterpenoids is just underway, a number of recent studies have identified key mechanisms that mediate the potent effects of triterpenoids. One of these mechanisms involves decreasing the levels of reactive oxygen species (ROS) through activation of Nrf2-dependent transcription [5,6]. In addition, the triterpenoids directly inhibit NF-κB signaling [7,8], a key pathway that regulates the production of a number of inflammatory mediators and their signaling cascades (e.g. TNF, IL-1β, IFNγ, TLR) [9]. Increased levels of antioxidant enzymes produced by Nrf2 reduce the cellular levels of ROS, thereby further attenuating NF-κB signaling and the transcription of pro-inflammatory genes such as iNOS and TNF [5,10,11].

The role of neuroinflammation in neurodegenerative disease has been under intense investigation in recent years and there is now overwhelming evidence that inflammation-induced oxidative stress compromises neuronal survival and may contribute to the progression of neurodegenerative diseases including Parkinson's (PD) and Alzheimer's disease (AD) (reviewed in [12-18]). We reasoned that if CDDO-Me were able to suppress microglial activities that contribute to neurotoxicity while promoting those that support neuronal survival, it may be capable of exerting neuroprotective effects. Therefore, the overall purpose of these studies was to investigate the cellular basis for the anti-inflammatory properties of CDDO-Me; specifically, to identify microglial activities modulated by CDDO-Me *in vitro *and the extent to which this modulation protects against inflammatory stimuli.

## Methods

### Animals

Experimental procedures involving use of animal tissue were performed in accordance with the NIH Guidelines for Animal Care and Use and approved by the Institutional Animal Care and Use Committee (IACUC) at The University of Texas Southwestern Medical Center in Dallas. Animals were housed in a climate controlled facility staffed with certified veterinarians.

### CDDO-Me

Lyophilized stocks of the synthetic triterpenoid CDDO-Me (RTA 402) were stored at -20°C until they were dissolved in DMSO.

### Cell culture

The murine BV2 microglia cell line was generated by Dr. Bistoni and colleagues by infecting primary microglial cell cultures with the v-raf/v-myc oncogene carrying retrovirus J2. These cells retain many of the morphological, phenotypical and functional properties described for freshly isolated microglial cells [19]. BV2 microglia were cultured in DMEM/F12 supplemented with 5% heat-inactivated fetal bovine serum (Sigma-Aldrich, St Louis MO), 1% penicillin-streptomycin, and 1% L-glutamine. The murine clonal hybrid cell line MN9D was developed by A. Heller and colleagues by somatic cell fusion of rostral mesencephalic tegmentum (RMT) from 14-day-old embryonic mice and the murine neuroblastoma cell line N18TG2 [20]. MN9D cells were grown in DMEM (Sigma-Aldrich) supplemented with 10% FBS (Gemini, West Sacramento CA), and 1% penicillin/streptomycin. To induce terminal differentiation of MN9D cells and increase their sensitivity to apoptotic stimuli, cells were incubated with 5 mM of valproic acid in N2 (Invitrogen, Carlsbad, CA)-supplemented serum-free DMEM for 3 days. Primary microglia were harvested from postnatal day 2–4 mouse pups using previously published protocols [21]. Briefly, brain tissue was removed, finely minced with a razor, incubated in a dissociation media containing 1 μL/mL DNAseI (Invitrogen), 1.2 U/mL dispase II (Roche), and 1 mg/mL papain (Sigma-Aldrich) in DMEM/F12 (Sigma-Aldrich) for 30–45 minutes at 37°C. After mechanical trituration, cells were centrifuged, passed through a 40 μM filter (BD Falcon, San Jose CA), and plated at 500,000 total cells/well in 6 well plates pre-coated with 0.1 mg/mL poly-D lysine (Sigma). Cells were fed every 3 days with fresh media (DMEM/F12 supplemented with 10% heat-inactivated fetal bovine serum (Sigma-Aldrich), 1% penicillin-streptomycin, and 1% L-glutamine). After 14–18 days *in vitro*, cultures were treated with 0.0625% trypsin-EDTA (diluted in DMEM/F12) for 45 minutes at 37°C to lift astrocytes and neurons from the wells, leaving a pure culture of primary microglia. The cultures were checked for purity and found to be greater than 95% microglia as measured by cell-type specific expression of CD68 and less than 5% astrocytes as measured by GFAP immunoreactivity. Murine peritoneal macrophages were obtained by eliciting an acute peripheral inflammatory reaction with intraperitoneal injection of thioglycolate [22]. Briefly, adult mice were injected intraperitoneally with 1 mL 3% Brewer's yeast thioglycolate. Three days later the animals were sacrificed, and 10 mL of cold sterile PBS (pH 7.4) was injected into the peritoneal cavity to wash out and recover peritoneal exudate. Cells were pelleted (1000 rpm, 5 min, 4°C), resuspended in culture media (high glucose DMEM supplemented with 10% fetal bovine serum (Atlanta Biologicals, Lawrenceville, GA), and 1% penicillin, streptomycin, and L-glutamine) and allowed to adhere to culture plates for 6 hr. Cells were washed twice with PBS to remove non-adherent cells and growth medium was replenished.

### Aggregation of amyloid beta peptide

Aβ 1–42 peptide was synthesized by Dr. Haydn Ball in the Protein Chemistry Core at UT Southwestern. Aggregation into fibrillar form was achieved by resuspending the peptide at final concentration of 100 μM into phosphate-buffered saline and incubating it at 37°C for 48 hrs. Thioflavin T fluorescence [23] and Congo Red binding *in vitro *was used to confirm fibril formation [24].

### Microglia proliferation and viability assays

Cell viability and proliferation were assayed in the BV2 microglia cell line using the Alamar Blue reagent (Invitrogen). Cells were seeded at 2000 cells/well in a 96-well plate. Cells were serum deprived for 3–5 hours, and CDDO-Me was added 1 hour before treatment with LPS as indicated. Alamar Blue was added as per the manufacturer instructions 2 hr before absorbance was read at 570 nm and 595 nm.

### Microglia activation assays

Rat embryonic ventral mesencephalon primary cultures were harvested from E14 pups, mechanically dissociated and seeded as micro-islands (25 uL of 1 × 10^6 ^cells/mL) on 4-chamber slides precoated with poly-D-lysine and laminin (BD Bioscience) in DMEM/F12 with 1% penicillin/streptomycin, glutamine, and non-essential amino acid and containing 10% fetal bovine serum (Atlanta Biologicals) and 10 ng/mL FGF-2 (R&D Systems, Minneapolis, MN). Culture media was changed after 2 days *in vitro *and cells were treated at day 5 *in vitro *with indicated compounds in DMEM containing 2.5% FBS and lacking FGF-2. Cells were fixed at 2 days post-treatment in 4% paraformaldehyde in PBS (pH 7.4) and stained with an antibody against activation marker F4/80 (1:60 dilution Serotec, Raleigh, NC) to quantify number of activated microglia. Each condition was done in triplicate; 20 random sites were visited per well; data was plotted as the average number of F4/80-positive microglia per field.

### Oligonucleotide microarrays

BV2 cells were plated at 500,000 cells/well in a 6-well plate in DMEM with 5% FBS and switched to serum-free media before pre-treatment with CDDO-Me or DMSO vehicle and subsequent stimulation with LPS as indicated. RNA was harvested as detailed below and levels of inflammation-related gene expression were detected on an oligonucleotide array as per manufacturer's instructions (Superarray Bioscience Corporation, Frederick, MD). Data analyses were performed using the Scatter Plot data analysis tool in the SuperArray GEArray Analysis Suite. The Scatter Plot displays the fold difference in the relative expression levels of genes between groups. Seven housekeeping genes were used for normalization. The control group was assigned to the X-Axis, and the treated group was assigned to the Y-Axis. An arbitrary boundary of 1.5-fold regulation in either direction was selected. If the fold increase was greater than boundary value, the gene names are shown in red with a plus (+) sign, and are located above the upper line. The further the sign is from the upper line, the greater the fold difference. If the fold decrease is greater than the boundary value, the genes are shown in green with a minus (-) sign, and are located below the lower line. The further the sign is from the lower line, the greater the fold difference. Black signs mean the fold change is not significant.

### Real time quantitative polymerase chain reaction

Real-time quantitative PCR (QPCR) was performed as previously described [25]. Briefly, total RNA was isolated from cultured cells or animal tissues using RNA Stat-60 (Tel-Test, Inc., Friendswood, TX), treated with DNase I (Invitrogen), and reverse transcribed using Superscript II RNase H-reverse transcriptase (Invitrogen). RNA concentration was determined by absorbance at 260 nm. Quantitative real-time PCR was performed using an ABI Prism 7900 HT Fast Detection System (Applied Biosystems Inc., Foster City, CA). Each 10 μl reaction was performed in 384-well format with 25 ng cDNA, 5 μl SYBR green PCR Master Mix, and 150 nM of each PCR primer. All reactions were performed in duplicate. Levels of mRNA expression were normalized to those of the mouse house-keeping gene cyclophilin B. Oligonucleotide primers for QPCR were obtained from Integrated DNA Technologies (Coralville, IA). The following mouse primers sequences were validated and used for gene amplification:

mTNF: forward 5'-CTG AGG TCA ATC TGC CCA AGT AC-3' and reverse 5'-CTT CAC AGA GCA ATG ACT CCA AAG-3'

mIL1β: forward 5'-CAA CCA ACA AGT GAT ATT CTC CAT G-3' and reverse 5'-GAT CCA CAC TCT CCA GCT GCA-3'

mMip1α: forward 5'-TTC ATC GTT GAC TAT TTT GAA ACC A-3' and reverse 5'-GCC GGT TTC TCT TAG TCA GGA A-3

iNOS: forward 5'-CAG GAG GAG AGA GAT CCG ATT TA-3' and reverse 5'-GCA TTA GCA TGG AAG CAA AGA-3'

### Multiplexed immunoassays

BV2 cells were plated at 500,000 cells/well in a 6-well plate in DMEM containing 5% FBS and switched to serum-free media before pre-treatment with CDDO-Me or DMSO vehicle and subsequent stimulation of LPS as indicated. Conditioned Medium (CM) was collected to measure the production of seven cytokines (IFN-γ, IL-1β, IL-6, IL-10, IL-12, KC/CXCL1, and TNF) using a multiplexed immunoassay per the manufacturer instructions (Meso-Scale Discovery, Gaithersburg, MD).

### Intracellular reactive oxygen species (ROS) imaging

Rat embryonic ventral mesencephalon neuron/glia cultures were prepared as published previously [26]. At 5 days *in vitro *(5 DIV), they were incubated with 3 μM DCFDA (Invitrogen) in serum-free growth medium for 40 min to quantify intracellular reactive oxygen species (ROS) production by fluorescence imaging. The next day, cells were treated with the vehicle, 1 μM fibrillar amyloid beta (Aβ) 42 peptide plus or minus 10 ng/mL LPS in the presence or absence of CDDO-Me as indicated for 24 hrs. Fluorescence images were captured on an Olympus CK40 microscope with a CoolSnap CCD ES monochromatic camera with a FITC filter in place. Quantification of fluorescence intensity was performed using intensity threshold analysis of digital images on an Alpha Innotech ChemiImager 4400 (Alpha Innotech, San Leandro, CA). MN9D dopaminergic cells were plated at 50,000 cells per well in DMEM containing 10% FBS in 24 well plates. The following day cultures were differentiated in N2 supplemented serum-free DMEM containing 5 mM valproic acid. 48 hours following differentiation MN9D cells were incubated with 3 uM DCFDA in serum free DMEM for 40 min, and then returned to differentiation media. Six hours after DCFDA loading, MN9D cells were treated with 10 nM CDDO-Me (or DMSO vehicle) for 16–20 hrs before a 30 minute stimulation with either TNF or conditioned media from LPS and CDDO-Me treated BV2 microglial cultures. BV2 microglial cultures for these experiments were plated in DMEM containing 5% FBS at 800,000 cells per well in a 6-well plate. Cultures were permitted to adhere to the plastic and then were pretreated with 10 nM CDDO-Me (or DMSO vehicle) for 16–18 hrs prior to stimulation with 10 ng/mL LPS (or saline vehicle). The following day conditioned media from BV2 cultures was removed and centrifuged at 1200 × g for 4 min before addition to DCFDA-loaded, differentiated MN9D dopaminergic cultures. Fluorescence images were captured on an Olympus CK40 microscope with a CoolSnap CCD ES monochromatic camera with a FITC filter in place. Quantification of ROS accumulation was performed by counting DCFDA-positive cell bodies in four fields per condition under 20× magnification (equivalent to approximately 50% of the plated area per well) in two independent experiments. DCFDA positive cells ranged between 38 and 420 per field depending on experiment and treatment. Values for treatment conditions in each experiment were expressed as fold increase in DCFDA positive cells per field relative to DMSO vehicle, saline treated control, and averaged between independent experiments.

### Cell survival/neuroprotection assays

MN9D dopaminergic cells (grown as described above), were terminally differentiated with 5 mM valproic acid in N2-supplemented serum-free DMEM 3 days prior to neuroprotection studies with CDDO-Me. MN9D cell viability was measured using the CellTiter 96 AQ_ueous _Assay reagent (Promega, Madison, WI). This reagent uses the tetrazolium compound (3-(4,5-dimethylthiazol-2-yl)-5-(3-carboxymethoxyphenyl)-2-(4-sulfophenyl)-2H-tetrazolium, inner salt; MTS) and the electron coupling reagent, phenazine methosulfate (PMS). MTS is chemically reduced into soluble formazan in metabolically active cells. MN9D cell viability was assayed by measurement of formazan absorbance at 492 nm in multi-titer 96-well plates at 492 nm during the last 2–4 hrs of a three-day incubation with soluble TNF or a two-day incubation with BV2 conditioned media in target-effector assays in which the BV2 microglia cell line was used as the effector cell and the MN9D dopaminergic ells as the target cell. Specifically, conditioned medium (CM) from LPS-treated BV2 microglia was transferred to MN9D cell cultures to induce inflammation-induced death in a dose-dependent manner.

### Phagocytosis assays

BV2 microglia were plated at a density of 50,000 cells/well in a 96-well plate and switched to serum-free media 24 hr later for stimulation as indicated with LPS (10 ng/mL) and/or fAβ (1 μM), in the presence or absence of CDDO-Me (10 nM). After 24 hr stimulation, phagocytosis was measured by exposing the cultures to fluorescently-labeled *E. coli *particles (Invitrogen) for 2 hr. Cells were incubated with trypan blue and rinsed with PBS to remove non-internalized particles prior to measuring fluorescence at 480 nm excitation and 520 nm emission on a Fluoroskan multiwell plate reader.

## Results

### CDDO-Me inhibits proliferation and activation of microglia

**Figure 1:**
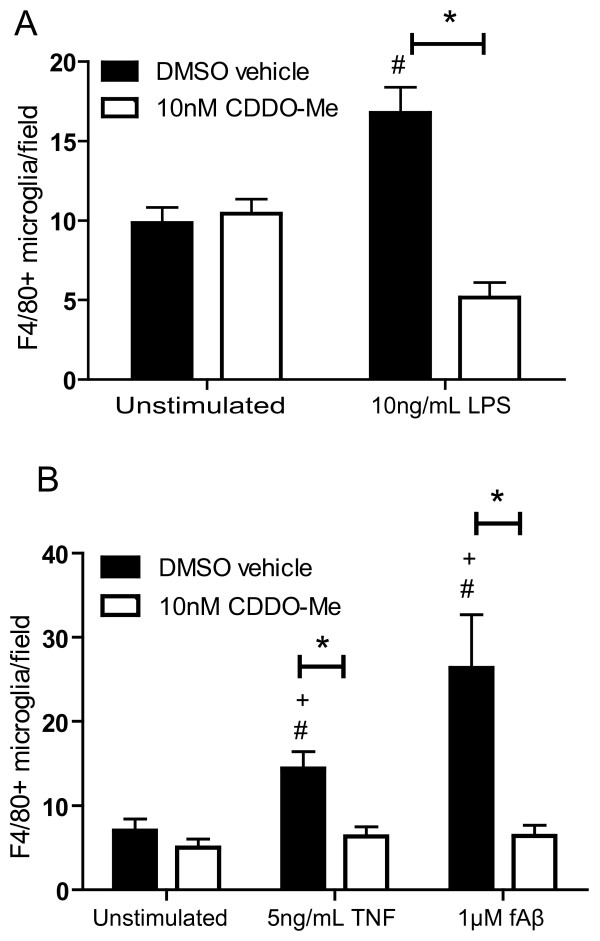
**CDDO-Me attenuates LPS-, TNF-, and fibrillar Aβ42-induced primary microglia activation.** Rat embryonic ventral mesencephalon primary cultures were treated at day 5 *in vitro *with indicated compounds (LPS 10 ng/mL, TNF 5 ng/mL, fAβ 1 μM, CDDO-Me 10 nM). Cells were fixed at 2 days post-treatment and stained with an antibody against the microglial activation marker F4/80. Values are expressed as mean number of F4/80-positive microglia per field ± S.E.M.. Values were analyzed by two-way ANOVA followed by Tukey post hoc test, * denotes CDDO-Me is significantly different from its DMSO vehicle for a given treatment; # denotes significant difference from the DMSO vehicle in unstimulated cells; + denotes significant difference from CDDO-Me in stimulated cells; all symbols at p < 0.05.

Neuroinflammatory processes are associated with a number of neurodegenerative conditions and have been implicated in the underlying progressive loss of neurons [15,16,27-30]. Therefore, inflammatory factors and mechanisms that contribute to neurotoxicity and compromise neuronal survival need to be elucidated and specifically targeted without interfering with the neuroprotective effects of glial activities [18]. Synthetic triterpenoids have potent anti-proliferative and differentiating effects on tumor cells and anti-inflammatory activities on activated macrophages. Therefore, we hypothesized that CDDO-Me may be able to promote neuronal survival by suppressing neurotoxic microglial activities without compromising overall microglial cell viability. In pilot experiments, we established the concentration range of CDDO-Me beyond which anti-proliferative and toxic effects could be detected in a murine BV2 microglial cell line. We found that CDDO-Me concentrations at or above 100 nM inhibited the proliferative response to bacterial lipopolysaccharide (LPS) as measured by Alamar blue reduction and that concentrations greater than 500 nM were toxic (data not shown). Based on these results, we chose to use concentrations of CDDO-Me at or below 100 nM for our studies. We first investigated the ability of CDDO-Me to inhibit activation of microglia in response to specific inflammatory stimuli. Treatment of rat primary mesencephalon neuron/glia cultures with CDDO-Me alone did not elicit microglia activation; but CDDO-Me pre-treatment resulted in attenuated LPS-, TNF- or fibrillar Aβ-induced increase in microglial activation, as measured by the number of F4/80-immunoreactive microglia (Fig. 1). These findings suggested that differential modulation of microglial activities might be possible at concentrations of CDDO-Me below 100 nM without cytotoxic effects to the cells.

### CDDO-Me suppresses transcription of inflammatory mediators in microglia and macrophages

**Figure 2:**
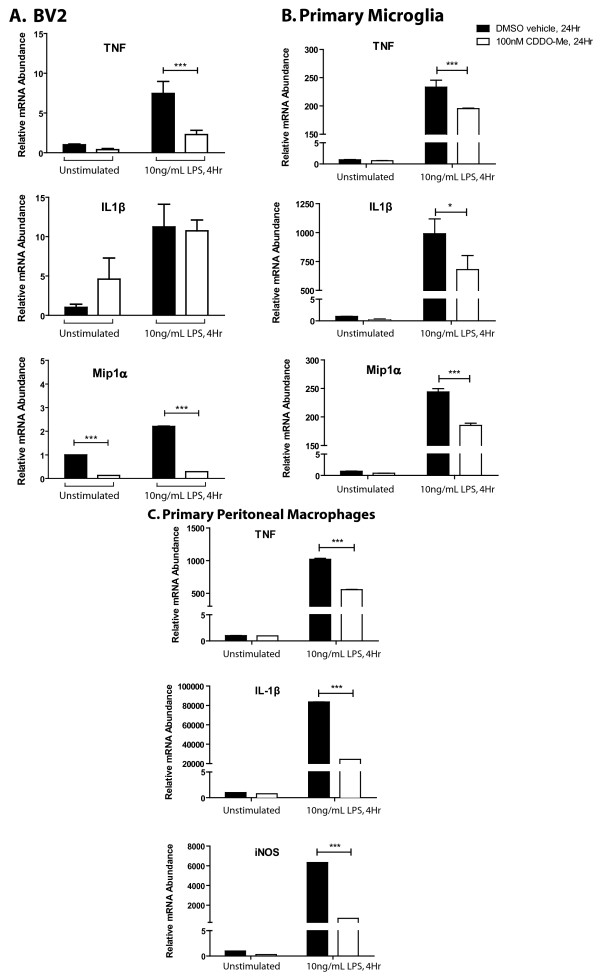
**CDDO-Me downregulates expression of mRNAs for pro-inflammatory genes.** (A) BV2 microglia were pre-treated with 10 nM CDDO-Me for 17 hr before a 4-hr stimulation as indicated. (B) Microglia from post-natal day 2 (P2) wild-type mice were pre-treated with 100 nM CDDO-Me for 24 hr before a 4 hr stimulation as indicated. (C) Peripheral macrophages from adult wild-type mice were pre-treated with 100 nM CDDO-Me for 24 hr before a 4-hr stimulation as indicated. Values represent mean ± S.E.M. and are expressed relative to vehicle-treated, vehicle-stimulated conditions. Values were analyzed by two-way ANOVA followed by Bonferroni's post hoc. * denotes significance at p < 0.05, *** denotes significance at p < 0.001.

**Figure 3:**
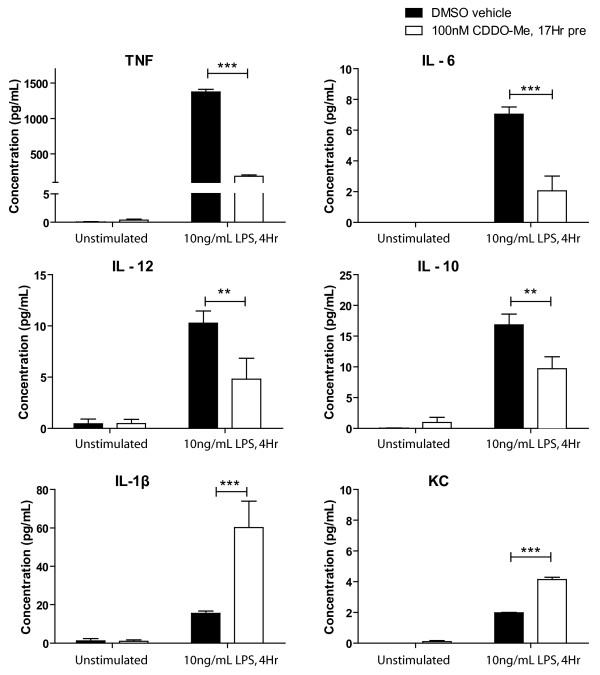
**CDDO-Me suppresses production of cytokines with known neurotoxic effects on dopaminergic cultures.** BV2 microglia were pre-treated with 100 nM CDDO-Me or DMSO vehicle for 17 hrs before a 4-hr stimulation with LPS (10 ng/mL). Inflammatory cytokine and chemokine production were measured from CM using a 7-plex inflammatory cytokine profile immunoassay from Meso-Scale Discovery on an MSD 2400 plate reader. Values represent mean cytokine production ± SEM. Values were analyzed by two way ANOVA followed by Bonferroni's post hoc. ** denotes significance at p < 0.01, *** denotes significance at p < 0.001.

A number of microglial-derived mediators have been reported to mediate neuronal death in experimental models of neurodegeneration (reviewed in [16]), raising the possibility that anti-inflammatory therapies may be an effective means of delaying or attenuating neuron death. Therefore, we investigated the ability of CDDO-Me to regulate expression of inflammation- and oxidative stress-related genes in primary microglia, macrophages, and the BV2 microglia cell line. We performed real-time quantitative polymerase chain reaction (QPCR) to measure mRNA levels for a number of inflammation-related genes. LPS-induced production of TNF mRNA was significantly reduced in all cell types while IL-1β mRNA was significantly reduced in primary microglia and macrophages but not BV2 cells (Fig. 2). CDDO-Me also significantly attenuated the LPS-induced mRNA expression of MIP-1α and iNOS. Multiplexed immunoassay analysis of BV2 microglia-conditioned medium (CM) confirmed that CDDO-Me attenuated LPS-induced production of factors such as IL-12, IL-6, and TNF all of which are known to promote autocrine signaling in microglia (Fig. 3). Given that both IL-6 and TNF can compromise DA neuron survival, these findings raised the possibility that CDDO-Me may be able to protect neuronal populations that display increased vulnerability to inflammation-induced oxidative stress and apoptotic death, in particular dopaminergic neurons [16,31], by limiting the production of these neurotoxic factors by chronically activated microglia without compromising other microglial functions. It should be noted, however, that not all chemokines and cytokines typically associated with pro-inflammatory responses were depressed by exposure to CDDO-Me. For instance, CDDO-Me increased production of CXCL1 (also known as keratinocyte-derived chemokine (KC)), a potent chemoattractant to neutrophils [32] (Fig. 3). Curiously, while CDDO-Me reduced (not significantly) LPS-induced IL-1β mRNA in the BV2 microglia cell line (Fig. 2), it potentiated secretion of this cytokine by BV2 cells (Fig. 3). The reason for this effect is unclear but these findings raise the interesting possibility that synthetic triterpenoids may be able to differentially regulate inflammatory responses by altering transcription of specific subsets of genes and perhaps by modulation of post-transcriptional signaling cascades that influence secretion of specific inflammatory factors. Future research into this area will provide much needed insight into other mechanisms of action for synthetic triterpenoids.

**Figure 4:**
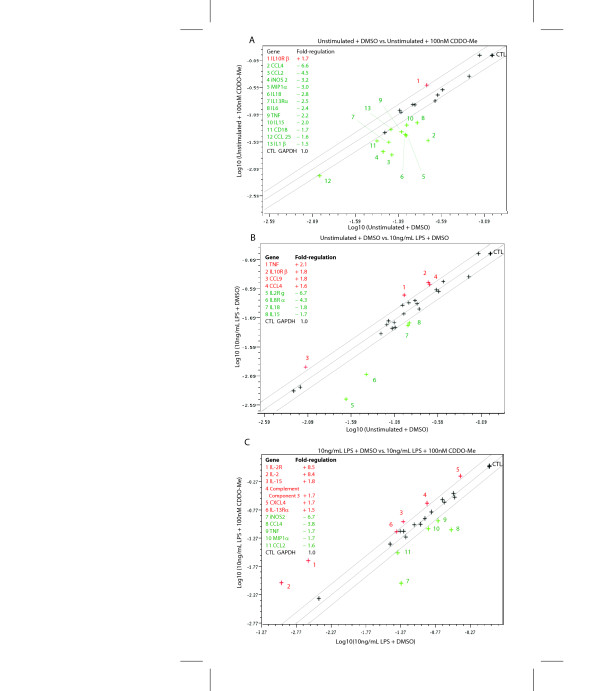
**CDDO-Me suppresses basal and inflammation-induced gene expression.** BV2 microglia cultures were pre-incubated with 100 nM CDDO-Me for 17 hrs before a 4-hr stimulation with 10 ng/mL LPS in serum-free medium. (A) Scatterplot of baseline inflammatory gene expression in unstimulated BV2 cells in the presence of DMSO vehicle (0.1%) versus unstimulated cells in the presence of 100 nM CDDO-Me. (B) Unstimulated cells in the presence of DMSO vehicle versus cells stimulated with LPS (10 ng/mL) in the presence of DMSO vehicle, or (C) cells stimulated with LPS in presence of DMSO vehicle versus cells stimulated with LPS in the presence of 10 nM CDDO-Me plus. Data analyses were performed using the Scatter Plot data analysis tool in the SuperArray GEArray Analysis Suite online (See Methods). Genes shown with a red plus (+) sign were up-regulated and genes shown with a green minus (-) sign were down-regulated in the treatment condition, which is plotted on the Y-axis relative to the control condition plotted on the X-axis.

To complement these findings and survey a larger number of inflammation-related genes, we performed oligonucleotide gene arrays in BV2 microglia treated with saline or LPS in the presence of CDDO-Me or its vehicle (DMSO). In resting microglia, CDDO-Me upregulated the receptor for a classic anti-inflammatory cytokine IL-10 while inhibiting basal expression of 12 inflammation-related genes, including TNF, iNOS2, IL-18, MCP1 (CCL2), Mip1α (CCL3), and Mip1β (CCL4) (Fig. 4a). These results indicate CDDO-Me coordinately attenuates gene expression of pro-inflammatory genes and enhances genes known to be part of anti-inflammatory responses. As expected, stimulation of BV2s with LPS triggered upregulation of the gene for the pro-inflammatory cytokine TNF as well as genes for the chemokines Mip1β (CCL4) and Mip1γ (CCL9) while downregulating IL-15, IL-18, IL-2, and IL-8 (Fig. 4b). Consistent with the well-known fact that the normal cellular response to an inflammatory stimulus is to activate anti-inflammatory response loops to return the cell to a pre-activation status, we also detected increased IL-10 receptor expression in response to LPS. In support of its anti-inflammatory properties, CDDO-Me attenuated LPS-induced increases in pro-inflammatory gene expression including TNF and MCP1 (CCL2) (Fig. 4c); these results are similar to those reported for CDDO-Im in LPS-stimulated peripheral neutrophils [6]. In agreement with real-time QPCR results, treatment with CDDO-Me blocked the LPS-induced increases in iNOS, MIP1α, and CCL2. Moreover, CDDO-Me was able to reverse the LPS-induced downregulation of IL-15 and IL-2 genes (Fig. 4c). Lastly, the oligonucleotide arrays indicated that expression of receptors for the anti-inflammatory cytokines IL-10 and IL-13 as well as complement component (required for destroying bacterial pathogens) were also upregulated by CDDO-Me exposure (Fig. 4b, c), suggesting CDDO-Me may be an effective anti-infective.

### CDDO-Me attenuates microglial-mediated neuronal oxidative stress

**Figure 5:**
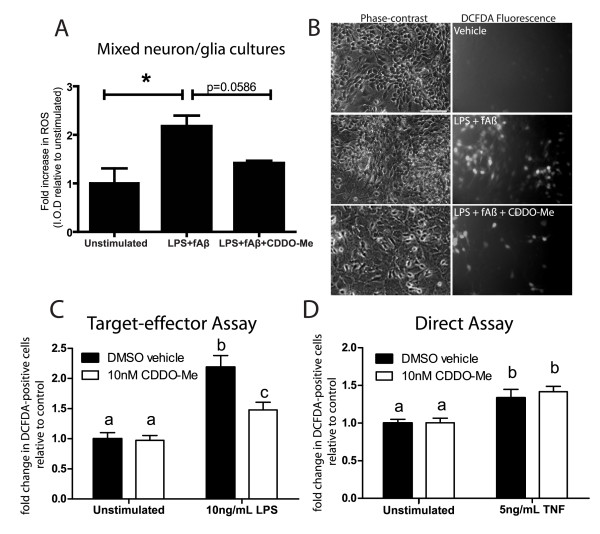
**CDDO-Me inhibits intracellular ROS accumulation induced by LPS and fibrillar Aβ42 in neuronal cultures from E14 rat basal forebrain cholinergic mixed neuron-glia cultures (BFCs) and in dopaminergic cells.** (A) Fold-change in intracellular ROS. Integrated optical density was measured from digital images of treated BFCs. Results are expressed as the mean ± S.E.M. of each condition relative to vehicle treatment. Values were analyzed by one-tailed Student's t-test, * denotes significance at p < 0.005. (B) BFCs loaded with the fluorescent indicator DCFDA. Intracellular ROS accumulation is evident after 24-hour treatment with 10 ng/mL LPS + 1 μM fAβ-42, but co-treatment with 100 nM CDDO-Me attenuated neuronal ROS accumulation. Scale bar = 50 μm. (C) Target-effector ROS accumulation assays of MN9D dopaminergic cells 30 minutes after transfer of conditioned medium (CM) from saline- or LPS-treated (24 hrs) BV2 microglia pre-incubated with CDDO-Me (10 nM) or vehicle (DMSO). (D) Direct treatment of MN9D dopaminergic cells with TNF (5 ng/mL) for 30 minutes after pre-incubation with CDDO-Me (10 nM) or vehicle (DMSO). (C, D) Values shown represent group means ± S.E.M of fold increase in DCFDA positive cell bodies relative to DMSO vehicle, saline treated control conditions and are averages of two independent experiments. Values were analyzed by two way ANOVA followed by Tukey's post hoc test. Groups denoted by different letters are significantly different at p < 0.05.

Oxidative stress, which is defined as the cellular condition when production of reactive oxygen and nitrogen species (ROS/RNS) exceeds the capacity of antioxidant defenses, is a trigger for glial activation and is a feature of most neurological and neurodegenerative conditions. Therefore, the use of antioxidants has been intensely investigated in models of neurodegeneration for their direct neuroprotective effects and for their ability to protect by suppressing the glial-mediated inflammatory response [33,34]. We investigated the ability of CDDO-Me to inhibit intracellular accumulation of ROS using the cell permeant dye DCFDA in mixed neuron-glia cultures from rat basal forebrain. Inflammation-induced intracellular ROS accumulation was evident primarily in cells with morphological characteristics consistent with that of neurons and to a lesser extent in the surrounding glial cells after treatment with LPS + fibrillar Aβ42 (Fig. 5a, b). Co-addition of CDDO-Me to these cultures inhibited the intracellular ROS accumulation induced by LPS + fibrillar Aβ42. The accumulation of ROS in neuronal cells was confirmed by treatment of the dopaminergic cell line MN9D with conditioned media from LPS and CDDO-Me treated BV2 microglial cells. Pretreatment of BV2s with 10 nM CDDO-Me before LPS exposure attenuated the ability of the conditioned media (CM) from BV2 microglia cultures to induce ROS accumulation in terminally differentiated MN9D cultures (Fig. 5c). Pretreatment of differentiated MN9Ds with CDDO-Me did not attenuate intracellular ROS accumulation resulting from direct TNF treatment (Fig 5d). Taken together, these findings suggest that the mechanism by which CDDO-Me protects neuronal cells may be primarily through changes in microglial-derived mediators and not through direct anti-oxidant effects on neuronal cells.

### CDDO-Me protects the dopaminergic MN9D cell line from inflammation-induced death

**Figure 6:**
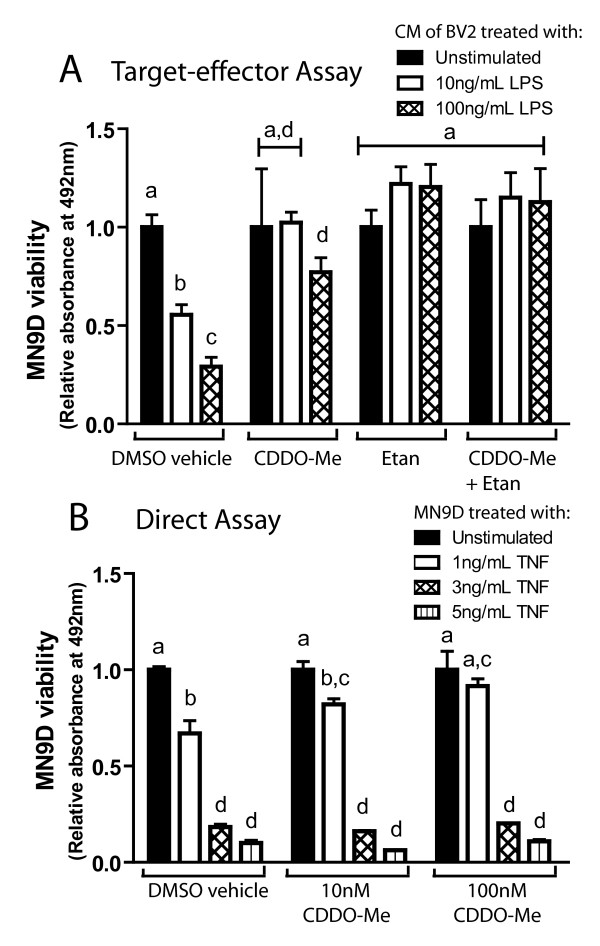
**CDDO-Me rescues dopaminergic MN9D cells by attenuating LPS-induced TNF production rather than by direct inhibition of TNF-induced death.** (A) MN9D cells were incubated with conditioned medium (CM) from BV2 microglia cultures stimulated with 0, 10 or 100 ng/mL LPS alone or with the same concentrations of LPS plus 10 nM CDDO-Me; 200 ng/mL etanercept; or both CDDO-Me and etanercept. Survival of MN9D cells was evaluated after 2 days of incubation in the CM using an MTS viability assay (See Materials and Methods). (B) Differentiated MN9D dopaminergic cells were treated with 0, 1, 3, or 5 ng/mL TNF alone or with the same concentrations of TNF plus 10 nM or 100 nM CDDO-Me. Survival was evaluated after 3 days using an MTS viability assay. Results are expressed as mean ± SEM. Values were analyzed by one-way ANOVA followed by Tukey's post hoc, groups denoted by different letters are significantly different from each other at p < 0.05.

Pro-inflammatory cytokines, in particular TNF, exert potent toxic effects on dopaminergic neurons and have been implicated in neurodegenerative disease pathogenesis [15,16,35,36]. To investigate the ability of CDDO-Me to protect dopaminergic neuron-like cells from inflammation-induced death, we performed *in vitro *target-effector cell survival assays. Survival of differentiated MN9D dopaminergic cells was measured after direct incubation with soluble TNF or in undifferentiated cultures after incubation with CM from LPS-treated BV2 microglia cells. We found that 10 nM CDDO-Me abolished MN9D cell death induced by exposure to CM from LPS-stimulated BV2 microglia (Fig. 6a), but ineffectively attenuated cell death induced by the direct addition of TNF to MN9D cells (Fig. 6b). These findings were consistent with a mechanism by which the neuroprotective effects of CDDO-Me in these assays are mediated by its ability to inhibit microglial-derived TNF production (Fig. 3) or by interfering with the intracellular site of action of other neurotoxic cytokines elicited by LPS rather than by direct inhibition of TNF-dependent death signaling by CDDO-Me. To test this idea directly, we added the TNF decoy receptor etanercept to bind and deplete soluble TNF from CM of BV2 microglia stimulated with LPS alone or in combination with CDDO-Me prior to CM transfer to MN9D dopaminergic cells. In support of this model, we found that etanercept attenuated MN9D cell death equivalent to that obtained with CDDO-Me treatment alone and together the two were not additive (Fig. 6a).

### CDDO-Me enhancement of microglial phagocytic activity is stimulus-specific

**Figure 7:**
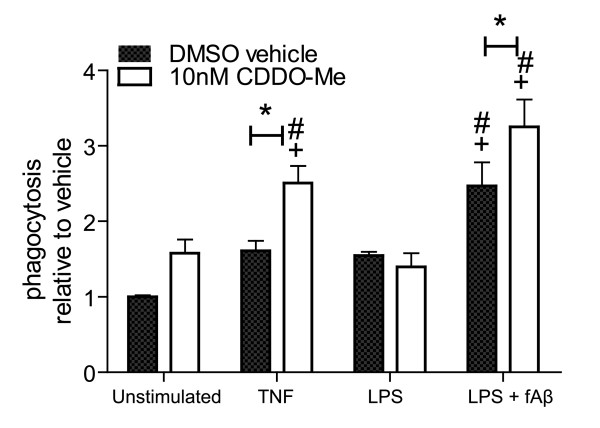
**CDDO-Me enhances phagocytic activity of BV2 microglia induced by TNF or LPS + fibrillar Aβ 1–42 peptide.** BV2 microglia were plated, switched to serum-free media 24 hours later, and stimulated overnight as indicated (LPS, 10 ng/mL; TNF, 10 ng/mL; fAβ42, 1 μM; and CDDO-Me, 10 nM.). Fluorescently-labeled *E. coli *particles were added to the cultures for 2 hours following stimulation and phagocytosis of particles was measured by fluorescence emission at 520 nm. Values represent mean phagocytic activity relative to baseline (vehicle-stimulated control) ± SEM. Values were analyzed by two-way ANOVA followed by Tukey's post hoc test, * denotes CDDO-Me is significantly different from its DMSO vehicle for a given treatment; # denotes significant difference from the DMSO vehicle in unstimulated cells; + denotes significant difference from CDDO-Me in stimulated cells; all symbols at p < 0.05.

Our findings that CDDO-Me attenuates production of some but not all inflammatory mediators raised the interesting possibility that other microglial activities could be modulated differentially by CDDO-Me. To investigate the ability of CDDO-Me to regulate phagocytic responses of BV2 microglia, we stimulated the cells with different inflammatory agents and analyzed their ability to phagocytose fluorescently labeled *E. coli *particles. We found that CDDO-Me had minimal effect on basal phagocytic activity of BV2 microglia or phagocytosis induced by LPS, but was able to enhance phagocytosis induced by TNF or fibrillar Aβ1–42 peptide (Fig. 7). These findings suggest stimulus-specific modulation of microglial phagocytosis by CDDO-Me and warrant further investigation in models of amyloid deposition.

## Discussion

Chronic neurodegenerative diseases are often associated with neuroinflammatory processes that may not only occur in response to neuron loss but may also contribute to it [15,16,27-30]. Because certain inflammatory responses in the brain are required for clearing cellular debris, limiting tissue damage, and contributing to wound repair, it is critical that inflammatory factors and mechanisms that contribute to neurotoxicity and compromise neuronal survival be identified and selectively targeted without interfering with the neuroprotective effects of glial activities [18]. Our previous studies established a critical role for TNF as a mediator oxidative neurotoxin- and endotoxin-induced dopaminergic neuron death in models of PD [26]. The ability of CDDO-Me to inhibit new synthesis of TNF in microglia and to effectively reduce soluble TNF production by activated cells offers one possible mechanism by which the anti-inflammatory properties of CDDO-Me affords protection to dopaminergic cells.

In addition to its anti-inflammatory properties, CDDO-Me may be able to boost or strengthen the immune system through upregulation of IL-2 signaling. IL-2 has been shown to be critical for survival, proliferation and differentiation of T-cells into effector cells and to confer a survival advantage to CD4+ T-cells to facilitate development of a memory population [37,38]. In chronic inflammatory syndromes characterized by persistent elevation of pro-inflammatory cytokines, localized IL-2 depletion at sites of inflammation has been shown to be the most profound effect of long term exposure to TNF [39]; our data suggests that therapeutic use of CDDO-Me may be able to reverse this phenomenon.

The observed antioxidant effects of CDDO-Me are not surprising given that synthetic triterpenoids have been shown to activate Nrf2, the key transcription factor that globally regulates the phase II detoxification pathway. Regardless of whether the primary antioxidant effect of CDDO-Me is mediated via direct action on neuronal populations or by suppression of glial-derived extracellular ROS production to reduce oxidative stress in neurons, the mechanisms by which synthetic triterpenoids exert antioxidant effects merit further investigation in animal models of neurodegeneration where oxidative stress is believed to be the primary mediator of neuron death. In support of this idea, it was recently reported that feeding pharmacological inducers of the phase II detoxification pathway to *Drosophila parkin *mutants or flies overexpressing α-synuclein suppressed the neuronal loss in both models of Parkinson's disease [40].

In summary, our findings indicate that in response to specific inflammatory triggers, CDDO-Me is able to differentially regulate microglial activities without compromising either microglial survival or the ability of microglia to perform basic functions (i.e. phagocytosis). Moreover, the ability of CDDO-Me to limit production and secretion of neurotoxic pro-inflammatory cytokines and to attenuate intracellular ROS accumulation strongly suggest that chronic administration of brain-permeant synthetic triterpenoids will confer neuroprotection *in vivo*. Several other anti-inflammatory agents have been reported to have neuroprotective properties in *in vitro *and *in vivo *models of Parkinson's disease. Specifically, the tetracycline derivative minocycline, which inhibits TNF synthesis, potently attenuates DA neuron loss resulting from nigral LPS treatment of rats [41]. Similarly, thalidomide (a non-selective immune modulating drug that reduces TNF expression through degradation of TNF mRNA) has been demonstrated to partially attenuate dopamine depletion in an MPTP mouse model of PD [42]. Naloxone, an opioid receptor antagonist, protected rat DA neurons against inflammatory damage through inhibition of microglia activation and superoxide generation [43]; and the kappa-opioid receptor agonist dynorphin A (1–17) attenuated inflammation-mediated degeneration of DA neurons in rat midbrain neuron-glia cultures [44]. In addition, dextromethorphan (DM), an ingredient widely used in antitussive remedies, has been shown to reduce the inflammation-mediated degeneration of DA neurons through inhibition of microglial activation [45]. Therefore, the neuroprotective properties of CDDO-Me and related compounds merit further investigation in pre-clinical animal models of PD.

Lastly, it may be of interest to determine the extent to which the CDDO-Me-induced enhancement of microglial phagocytic activity observed in our *in vitro *studies can be achieved *in vivo *with brain-permeant synthetic triterpenoids. This issue may be of particular therapeutic relevance in the treatment of AD because pro-inflammatory cytokines, including TNF, have been shown to preferentially attenuate phagocytic activity of microglia induced by fibrillar Aβ amyloid peptides (but not by IgG antibody activation of Fc Receptor) through an E prostanoid receptor-dependent mechanism [46]. On the basis of our results, we speculate that synthetic triterpenoids will be able to promote fibrillar amyloid clearance by potentiating the phagocytic activity of microglia at plaque sites characterized by inflammation where TNF is locally elevated. If synthetic triterpenoids can promote plaque clearance, their use as an adjunct therapy to reduce amyloid burden in patients with AD may be beneficial.

## Competing interests

TAT, MKM, and MGT have no competing interests. MBS receives grant support from Reata Pharmaceuticals.

## Authors' contributions

TAT performed the proliferation assays, gene expression studies, multiplexed immunoassays, statistical analysis, and helped draft the manuscript. MKMcC performed the microglia activation, phagocytosis, neuroprotection assays, ROS imaging assays, established primary cultures, statistical analysis, and helped draft the manuscript. MGT performed the ROS imaging studies, conceived and designed the study, and drafted the manuscript. MBS provided reagents and critical input during drafting of the manuscript. All authors read and approved the final manuscript.
